# Association between Social Determinants of Health and Telehealth Use Among Older Adults

**DOI:** 10.1093/geroni/igaf122.2792

**Published:** 2025-12-31

**Authors:** Misun Hwang, Philip Veliz, Yun Jiang

**Affiliations:** University of Michigan, Ann Arbor, Michigan, United States; University of Michigan, Ann Arbor, Michigan, United States; University of Michigan, Ann Arbor, Michigan, United States

## Abstract

Telehealth holds promise to enhance health outcomes for older adults, yet significant disparities remain in access and utilization. Although examining social determinants of health (SDOH) could provide valuable insights to bridge the digital divide, little is known about how these factors affect telehealth use. This study explored the association between SDOH and telehealth use among older adults in the United States. Using logistic regression, we analyzed data from the 2023 National Health and Aging Trend Study (NHATS), a nationally representative survey including 8,597 older adults (≥65 years). According to the Healthy People 2030 SDOH framework, five dimensions of SDOH were assessed: economic stability, education, healthcare access and quality, social context, and neighborhood and built environment. Telehealth use was measured by communication with a provider, insurance handling, or health information access using the Internet or online platforms (yes/no). Findings revealed that over half of older adults (50.3%) used telehealth in the past year. Those with less than a college education (OR: 0.28, CI: 0.24-0.32), living in rural areas (OR: 0.59, CI: 0.44-0.81), lacking social networks (OR: 0.48, CI: 0.32-0.72), or with neighborhood safety concerns (OR: 0.58, CI: 0.45-0.75) were less likely to use telehealth. Economic stability (measured by food insecurity, home ownership, and ability to pay utilities) was not associated with telehealth use after controlling for other factors. While multiple dimensions of SDOH significantly affected telehealth use among older adults, addressing the impact of these factors in telehealth services and providing tailored support will improve older adults’ engagement and health outcomes.

